# The relationship between sonographically assessed volumetric brain development in VLBW preterm infants and neurodevelopmental outcome at 2 years of age—data from the NeoNEVS project

**DOI:** 10.3389/fped.2026.1769403

**Published:** 2026-04-13

**Authors:** C. Brickmann, R. Lampe, I. Sidorenko, N. Gauger, J. Hauer, M. Krüger, S. Loth

**Affiliations:** 1 Department of Neonatology, TUM University Hospital, Technical University of Munich, TUM School of Medicine and Health, Munich, Germany; 2 Clinic for Neonatology, Muenchen Klinik GGmbH, Munich, Germany; 3 Department of Clinical Medicine, Center for Digital Health and Technology, Orthopedic Department, Research Unit for Paediatric Neuroorthopedics and Cerebral Palsy of the Buhl-Strohmaier Foundation, Klinikum Rechts der Isar, School of Medicine and Health, Technical University of Munich, Munich, Germany; 4 Markus Würth Professorship, Technical University of Munich, Munich, Germany; 5 TUM School of Medicine and Health, Department of Pediatrics, Kinderklinik Muenchen Schwabing, TUM University Hospital, Technical University of Munich, Munich, Germany

**Keywords:** Bayley III = bayley scale of infant development, brain growth, cerebral ultrasound, neurodevelopment, VLBW (very low birth weight)

## Abstract

**Background:**

Very low birth weight (VLBW) preterm infants are at increased risk for long-term neurodevelopmental impairment. Early identification of infants at risk remains challenging, particularly with regard to dynamic brain development during neonatal intensive care. Cranial ultrasound (CUS) allows safe and repeated bedside assessment of cerebral growth over time.

**Methods:**

In this retrospective cohort study, 79 VLBW infants (<1,500 g, <32 weeks gestation) treated at two tertiary neonatal intensive care units between 2019 and 2021 were included. Serial cranial ultrasound examinations were performed from birth to discharge. Total brain volume was estimated using a validated ellipsoid model, and individual cerebral growth rates were derived from longitudinal measurements. Neurodevelopmental outcome at 24 months corrected age was assessed using the Bayley Scales of Infant and Toddler Development, Third Edition (Bayley-III), reporting percentile ranks for cognitive, language, motor, and a combined developmental score. Associations were evaluated using Spearman correlation and multivariable linear regression.

**Results:**

The median cerebral brain growth rate was 2.4 cm^3^/day (95% CI: 1.5–3.1). Cerebral growth rate demonstrated a modest but statistically significant positive correlation with Bayley-III motor percentile rank (*r* = 0.25, *p* < 0.05). Associations with cognitive, language, and combined Bayley outcomes were positive but did not reach statistical significance. Total brain volume at discharge was not associated with neurodevelopmental outcomes in any domain. Cerebral growth rate was modestly correlated with the decline in head circumference percentile from birth to discharge. Conclusion: Longitudinal ultrasound-derived cerebral brain growth is associated with motor development at two years corrected age in VLBW infants, whereas single time-point brain volume measurements are not. Serial cranial ultrasound represents a feasible bedside approach to complement clinical risk assessment and may contribute to early neurodevelopmental risk stratification.

## Introduction

Preterm birth remains a major global health concern, contributing to significant neonatal morbidity and long-term neurodevelopmental impairment. Among this population, very low birth weight (VLBW) infants represent a particularly vulnerable group due to the immaturity of brain structures and heightened susceptibility to both injury and disrupted brain growth (1, 2). Despite improvements in neonatal intensive care and survival rates, the risk of cognitive, motor, and behavioral difficulties in this group remains substantial (3, 4).

Neurodevelopment in VLBW infants is influenced by a complex interplay of perinatal factors including gestational age (GA), birth weight, systemic illness, mechanical ventilation, and exposure to inflammation or hypoxia (5, 6). These exposures, occurring during critical windows of brain development, are thought to disrupt both macro- and microstructural brain maturation (7–11). Early detection of neurodevelopmental risk through bedside imaging biomarkers could enable timely intervention and improve long-term outcomes.

Cranial ultrasonography (CUS) is the most accessible and widely used neuroimaging modality in neonatal units. Its portability, safety, and low cost make it an ideal tool for serial assessment of brain development, especially in resource-constrained settings (12, 13). Traditionally, CUS has been employed for detecting major structural abnormalities such as intraventricular hemorrhage (IVH) and periventricular leukomalacia (PVL), which are known to correlate with adverse outcomes (14, 15). However, increasing attention is now being paid to the potential of CUS for quantitative assessment of brain growth trajectories, offering dynamic insight into cerebral development beyond static injury classification (16, 17).

Emerging evidence indicates that longitudinal changes in cerebral dimensions—such as biparietal diameter (BiPD), interhemispheric distance, and estimated brain volume—can be reliably tracked using standardized 2D and 3D ultrasound protocols (18–20). These ultrasound-based metrics have shown correlation with head circumference growth (21, 22), Magnetic Resonance Imaging (MRI)-based brain volume estimates (23) and, importantly, later neurodevelopmental outcomes. Notably, multiple studies have highlighted that the trajectory of brain growth—rather than a single measurement at term-equivalent age—may offer more accurate predictive value for cognitive and motor development (24, 25).

Structural brain measurements obtained at term-equivalent age have been consistently associated with later neurodevelopmental outcomes. Total cerebral volume measured by MRI has shown significant correlations with cognitive and motor performance at two years corrected age, while specific linear parameters such as BiPD and interhemispheric distance have been identified as predictors of developmental scores as early as six months. However, the utility of such findings is limited by the common reliance on single-time-point MRI assessments. In very preterm and VLBW infants, repeated MRI examinations are often not feasible due to the clinical instability of these patients. The need for transport, extended positioning, temperature management or sedation introduces potential risks that make serial MRI logistically challenging in this particularly vulnerable population (23, 26–28).

In contrast to MRI measurements, CUS provides a practical, noninvasive, and repeatable imaging modality that can be implemented routinely throughout the neonatal period. Evidence from ultrasound-based studies supports its prognostic utility, demonstrating that quantitative measures of brain development obtained at the bedside can reflect later neurodevelopmental outcomes. For instance, elevated ventricular-to-brain ratios have been linked to an increased risk of mental delay by two years of age (12–14, 29). Additionally, abnormal trajectories of cerebral growth observed through serial ultrasound assessments have been significantly associated with lower cognitive and psychomotor developmental scores. These findings underscore the potential of longitudinal cranial ultrasound, when applied systematically and interpreted quantitatively, to serve as a valuable alternative to advanced neuroimaging for predicting long-term neurodevelopmental trajectories in preterm infant (22, 30).

These insights support the hypothesis that longitudinal monitoring of brain growth using CUS could serve as a low-cost, high-impact screening tool for early identification of infants at risk for poor neurodevelopmental outcomes. Yet, despite promising findings, many existing studies focus either on isolated anatomical measures (e.g., ventricular size or cerebellar diameter) or rely on cross-sectional data at term age, missing the dynamic developmental processes unfolding in the preterm period (31–33). Moreover, very few studies have evaluated the relationship between serial sonographic assessments and Bayley-III scores at 24 months, a validated and clinically meaningful endpoint.

Bayley-III remains a widely used standardized tool to assess early cognitive, motor, and language development in high-risk populations, including preterm infants (33, 34). Studies show that performance of Bayley-III on functional domains (cognitive, motor, and language) at 24 months is associated with later school-age IQ, academic achievement, and behavioral outcomes (35). Percentile-based outcomes allow clinically intuitive interpretation of developmental performance and facilitate comparison across cohorts. However, the predictive value of early imaging for Bayley outcomes varies depending on the modality, timing, and type of analysis used (3, 9). Thus, there is a need for robust, longitudinally designed studies that bridge this imaging-outcome gap.

Our study focuses on the correlation between sonographically assessed longitudinal brain growth in VLBW infants and their neurodevelopmental outcome at 2 years corrected age and test for robust correlations between brain growth trajectories and developmental outcomes.

## Methods

### Study design and population

This retrospective cohort study included consecutively treated preterm neonates born at less than 32 weeks of gestational age with a birth weight below 1,500 grams. All infants were cared for in two tertiary-level (Level III) neonatal intensive care units in Bavaria, Germany, between January 2019 and December 2021. The initial cohort comprised 194 infants. Exclusion criteria were conditions known to substantially affect cerebral development, including intraventricular or intracerebral hemorrhage exceeding Papile grade II (35), cystic periventricular leukomalacia, hydrocephalus, congenital brain malformations, death prior to hospital discharge, interfacility transfers (outborn infants), and incomplete or inconsistent clinical or imaging data. After exclusions, 153 infants remained eligible. Of these, 79 infants completed standardized neurodevelopmental follow-up at 24 months corrected age using the Bayley Scales of Infant and Toddler Development, Third Edition (Bayley-III), and were included in the final analysis.

### Cranial ultrasound acquisition and brain volume estimation

Cranial ultrasound (CUS) examinations were performed by trained neonatologists at both participating NICUs using standardized imaging equipment (Philips Affiniti 70 ultrasound system with a Philips C 8–5 MHz broadband microconvex transducer). Ultrasound assessments were conducted as part of routine clinical care in accordance with institutional protocols and national guidelines. Standardized examination time points included days of life 1, 3, 7, 14, and 28, followed by biweekly intervals until discharge, with additional scans obtained as clinically indicated. Each examination was performed on a single day of life, with one measurement set obtained per examination day; no repeated measurements within the same day of life were included. All ultrasound examinations performed during the NICU admission were included in the longitudinal analysis, and the repeated within-subject structure of the data was accounted for using linear mixed-effects modelling.

**Figure 1:**
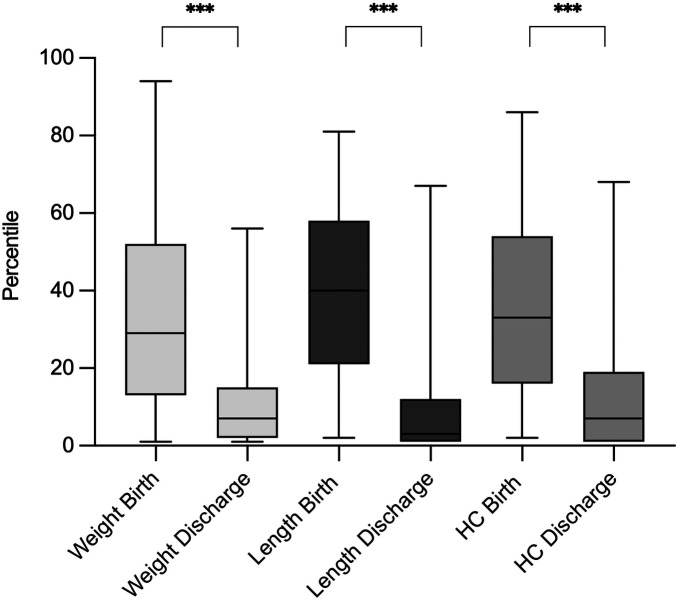
Change in body composition percentiles regarding weight, length and head circumference from birth to discharge. HC, head circumference. ***p < 0.001.

All examinations adhered to standardized neurosonographic guidelines issued by the German Society for Ultrasound in Medicine (DEGUM), ensuring consistent acquisition of coronal and sagittal planes. Retrospective image analysis was performed using the institutional Picture Archiving and Communication System (Centricity Universal Viewer Zero Footprint, GE Healthcare, Chicago, IL, USA), enabling precise linear measurements on static ultrasound images. Total brain volume (TBV) was estimated using a geometric ellipsoid model based on three orthogonal parenchymal dimensions: biparietal diameter (BiPD), anteroposterior axis (APA), and vertical axis (VA), according to the formula (Figure 1):

TBV=(4/3)×π×(BiPD/2)×(APA/2)×(VA/2)

This approach has been previously validated (36). All measurements were recorded in millimeters, and calculated volumes were expressed in cubic centimeters (cm^3^). BiPD was measured in the third coronal plane as the maximal lateral distance between the outer margins of the parietal cortex. APA and VA were obtained from the midline sagittal plane, with APA defined as the fronto-occipital length along a horizontal axis through the corpus callosum and VA defined as the maximal craniocaudal distance from the cortical surface beneath the transducer to the foramen magnum, identified by distinct echogenic landmarks.

### Inter- and intra-observer reliability

Each measurement set was independently reviewed by a second neonatologist with specialized training in neonatal neurosonography. Measurement reliability was assessed using intraclass correlation coefficient (ICC) analyses. Intra-observer repeatability was evaluated using a two-way mixed-effects model with absolute agreement for single measurements (ICC[3,1]), and inter-observer agreement was assessed using a two-way random-effects model with absolute agreement (ICC[2,1]), both reported with 95% confidence intervals. Reliability analyses were performed separately for each linear measurement dimension and for derived TBV, based on 82 paired measurements from 15 additional infants not included in the primary cohort.

### Clinical and neurodevelopmental data collection

Demographic and clinical data were extracted from electronic medical records and included gestational age, birth weight, sex, and body composition parameters at birth and discharge. Neonatal comorbidities documented during hospitalization included patent ductus arteriosus (PDA), bronchopulmonary dysplasia (BPD), sepsis, necrotizing enterocolitis (NEC), focal intestinal perforation (FIP), and intraventricular hemorrhage classified as Papile grade I or II. Respiratory support variables included the incidence and cumulative duration of invasive mechanical ventilation (IMV) and non-invasive ventilation (NIV).

Neurodevelopmental outcome was assessed at 24 months corrected age using the Bayley Scales of Infant and Toddler Development, Third Edition (Bayley-III), which evaluates cognitive, language (receptive and expressive), and motor (fine and gross) development. Percentile ranks relative to age-matched normative data were used as the primary outcome measures (37, 38). To obtain a global developmental metric, a combined Bayley percentile score was calculated by averaging percentile ranks across the three domains.

### Statistical analysis

Statistical analyses were performed using IBM SPSS Statistics version 27 (IBM Corp., Armonk, NY, USA) and GraphPad Prism version 10 (GraphPad Software, San Diego, CA, USA). Continuous variables were analyzed using the Wilcoxon rank-sum test, and categorical variables were summarized descriptively. Unadjusted associations between brain growth parameters and Bayley-III percentile outcomes were assessed using Spearman's rank correlation coefficients with two-sided testing. Correlation strength was interpreted descriptively. Multivariable linear regression analyses were performed to evaluate independent associations between cerebral brain growth and neurodevelopmental outcomes, adjusting for covariates selected *a priori* based on established clinical relevance and to minimize collinearity among growth-related variables.

Longitudinal brain volume development was analyzed using linear mixed-effects models to account for repeated measurements and within-subject correlations, allowing estimation of fixed and random effects and characterization of individual growth trajectories during hospitalization.

Given the retrospective design, no *a priori* sample size calculation was performed. A post-study assessment indicated that with 79 participants, the study was powered to detect correlation coefficients of approximately *r* ≥ 0.31 with 80% power at a two-sided *α* level of 0.05. For all analyses, a two-sided *p*-value <0.05 was considered statistically significant.

## Results

### Cohort characteristics

A total of 79 VLBW preterm infants were included in the final analysis. Median gestational age at birth was 28 + 5 weeks (IQR: 27 + 1–29 + 6), and median birth weight was 1,040 g (IQR: 850–1,240 g). Baseline demographic and clinical characteristics, as well as body composition parameters at birth and discharge, are summarized in Table 1. Females constituted 61% of the cohort, and the majority of infants were delivered by cesarean section (96%). Common neonatal comorbidities included sepsis (30%), patent ductus arteriosus (23%), and bronchopulmonary dysplasia (14%). All infants required non-invasive respiratory support, and 43% received invasive mechanical ventilation. Only mild intraventricular hemorrhage (Papile grade I–II) was present (10%), consistent with inclusion criteria. These and Bayley-Test demographic data are visualized in Table 1.

**Table 1:** Demographic cohort data. GA, gestational age; IQR, interquartile range; Perc., percentile; UC, umbilical artery; IMV, invasive mechanical ventilation; NIV, non-invasive ventilation; PDA, patent ductus arteriosus; BPD, bronchopulmonary dysplasia; AIS, amnion infection syndrome; EOS, early onset sepsis; LOS, late onset sepsis; BC, blood culture; NEC, necrotizing enterocolitis; FIP, focal intestinal perforation; IVH, intraventricular hemorrhage; ***p < 0.001 testing Birth to Discharge.

Demographic data	Birth		Discharge	
*N* = 79	Median	IQR 25/75/%	Median (*p*-value)	IQR 25/75/%
GA (week + day)	28 + 5	27 + 1/29 + 6		
Weight (g)	1,040	850/1,240	2,330	2,090/2,570
Weight Perc.	29	13/52	7 (***)	2/15
Length (cm)	37	35/39	45	43/46
Length Perc.	40	21/58	3 (***)	1/12
Head circumference (cm)	26	24/28	32	31/33
Head circumference Perc.	33	16/54	7 (***)	1/19
APGAR 1	6	5/8		
APGAR 5	8	7/9		
APGAR 10	9	8/10		
pH UA	7.3	7.3/7.4		
IMV (h)	0	0/72		
NIV (h)	744	336/1,176		
IMV (Yes/No)	34/45	43/57		
NIV (Yes/No)	79/0	100/0		
	Overall number	%	Number in Category	%
Sex (male/female)	31/48	39/61		
Birth Mode (spontaneous/Cesarean Section)	3/76	4/96		
PDA	18	23		
None		61	77	
Medical Treatment		17	22	
Surgical Treatment		1	1	
BPD	11	14		
None		68	86	
Mild		6	8	
Moderate		3	4	
Severe		2	2	
Sepsis	24	30		
None		55	69	
AIS		3	4	
EOS BC negative		0	0	
EOS BC positive		3	4	
LOS BC negative		7	9	
LOS BC positive		11	14	
NEC/FIP	4	5		
None		75	95	
Medical Treatment		1	1	
Surgical Treatment		3	4	
IVH	8	10		
None		71	90	
I		7	9	
II		1	1	

### Inter- and intra-observer reliability

Sonographic measurements demonstrated excellent repeatability and agreement. Intra-observer reliability analysis based on 82 paired measurements from 15 infants showed high consistency across all linear dimensions and derived total brain volume, with intraclass correlation coefficients (ICCs) ranging from 0.94 to 0.98. Inter-observer agreement was similarly robust, with ICCs between 0.92 and 0.97 for linear dimensions and between 0.97 and 0.98 for total brain volume (see Supplementary Material).

### Postnatal somatic growth

**Figure 2:**
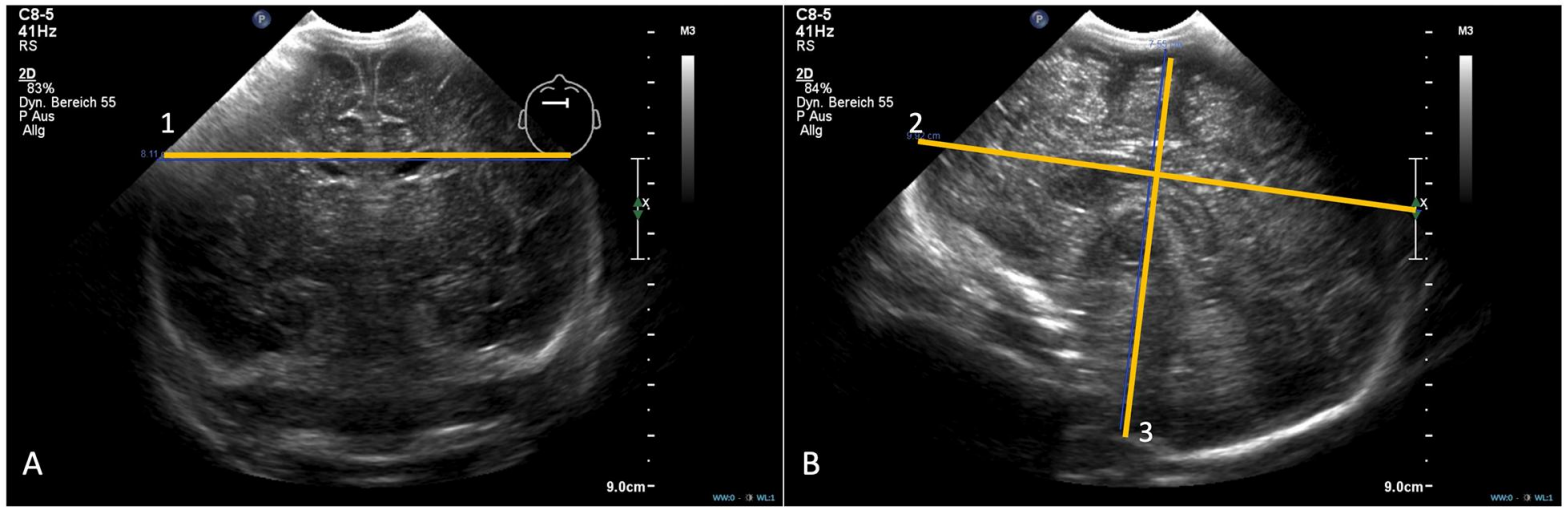
Examples of the coronar **(A)** and sagittal planes **(B)** including the biparietal diameter (1), anterioposterior axis (2) and vertical axis (3).

Between birth and discharge, weight, length, and head circumference increased in absolute terms, while their respective percentile ranks declined significantly (Table 1, Figure 2). Median weight percentile decreased from 29 to 7, length percentile from 40 to 3, and head circumference percentile from 33 to 7. The decline in percentile ranks was statistically significant for all parameters (*p* < 0.001).

### Cerebral volume growth

**Figure 3:**
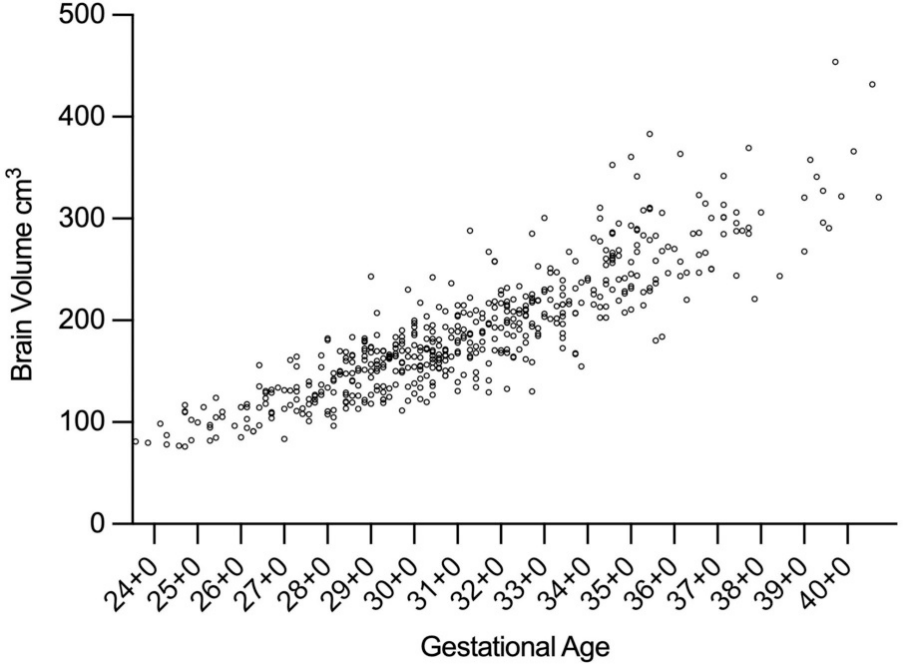
Scatterplot of the longitudinal brain volume measurements of all patients. Abr.: GA, Gestational Age.

Serial cranial ultrasound assessments combined with linear mixed-effects modelling demonstrated a median cerebral volume growth rate of 2.4 cm^3^/day (95% CI: 1.5–3.1), described in Table 2. Individual growth trajectories are illustrated in Figure 3.

**Table 2:** Descriptive data of Bayley-III test results, brain growth, brain volume at discharge and the. Percentile Drop of HC at birth compared to Discharge. To present a clearer interpretation of the dynamics the change is presented using percentiles rather that absolut measurement which would all be higher at discharge than at birth.

Descriptive data of:	Mean/*n* = (%)	Std. dev.
Bayley cognitive	69.2	31.4
Bayley language	52.9	33.4
Bayley motor	65.5	25.7
Bayley combined	62.6	26.7
Brain growth (cm3/d)	2.38	0.39
Brain volume at discharge (cm^3^)	279.7	53.4
Head circumference percentile drop	−23.4	22.1

### Associations between brain growth and bayley-III outcomes

**Figure 4:**
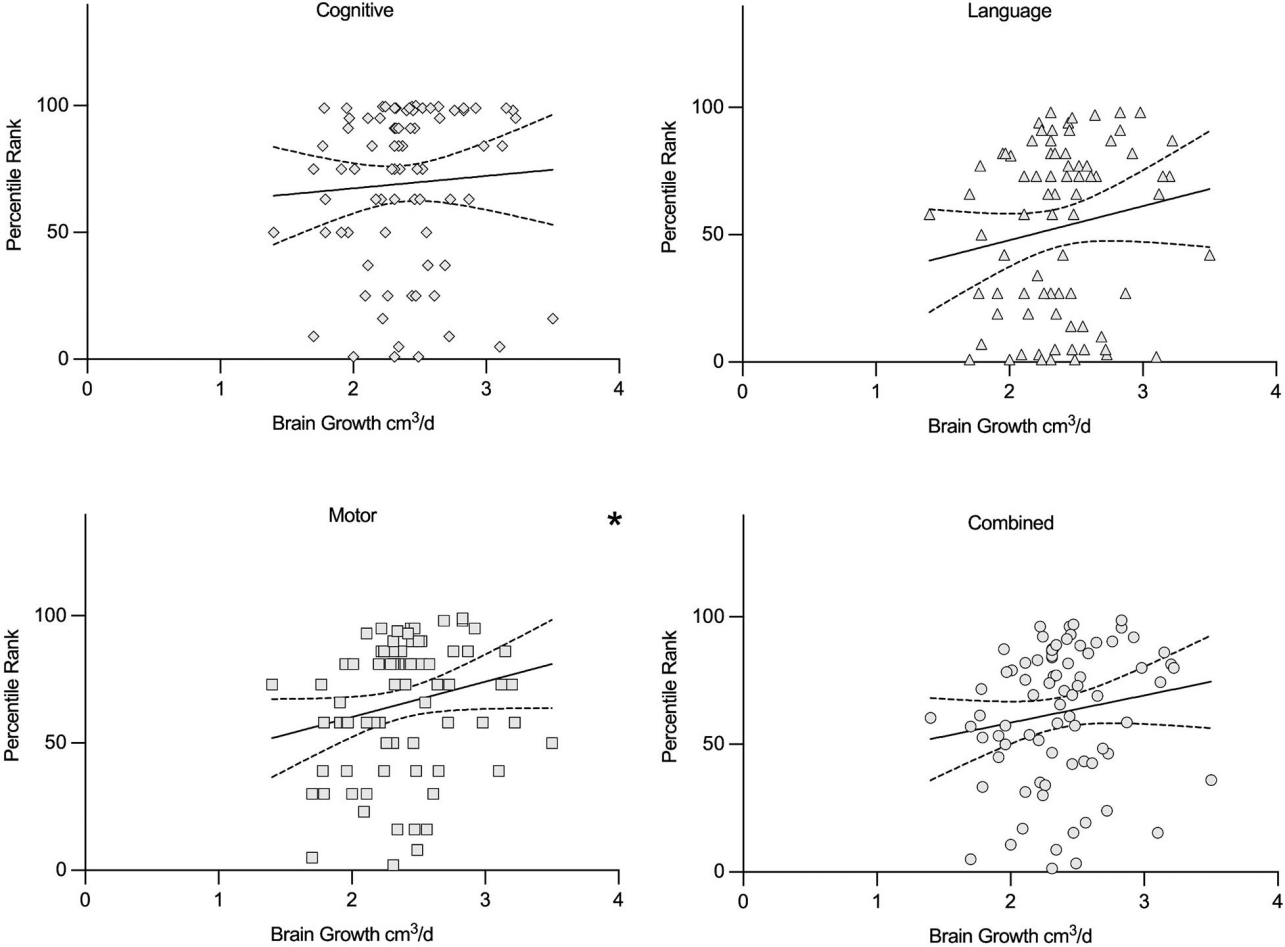
Scatterplots illustrating the association between cerebral growth rate (cm^3^/day) and Bayley-III percentile ranks for cognitive, language, motor, and combined developmental domains. A statistically significant positive correlation was observed for the motor domain only (*r* = 0.25, *p* < 0.05). Regression lines with 95% confidence intervals are shown for visual orientation and are not intended to represent the underlying Spearman rank correlation analysis. *p < 0.05. HC, head circumference.

Unadjusted associations were evaluated using Spearman correlation analyses. A modest but statistically significant positive correlation was observed between cerebral growth rate and Bayley-III motor percentile rank (*r* = 0.25, *p* < 0.05). Positive but non-significant correlations were observed for the language (*r* = 0.17), cognitive (*r* = 0.13) and combined (*r* = 0.19) domains. These results are summarized in Table 2 and visualized in Figure 4.

### Head circumference percentile change and brain growth

**Figure 5:**
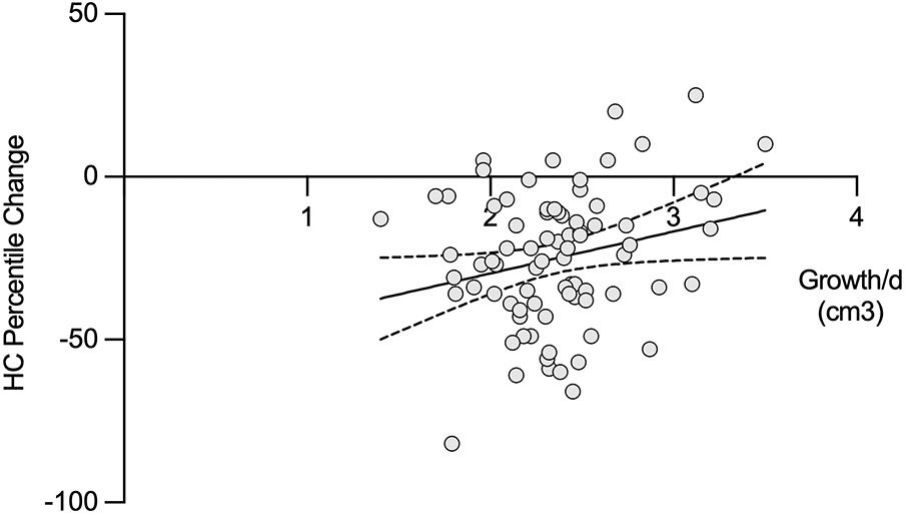
Scatterplot illustrating the association between cerebral growth rate (cm^3^/day) and the change in head circumference percentile from birth to discharge. A weak but statistically significant positive correlation was observed (*r* = 0.16, *p* < 0.05). Regression line with 95% confidence interval is shown for visual orientation only. HC, head circumference. *p < 0.05.

Cerebral growth rate showed a weak but statistically significant positive correlation with the decline in head circumference percentile from birth to discharge (*r* = 0.16, *p* < 0.05; Figure 5), indicating a statistically detectable but clinically modest relationship. No significant association was observed between head circumference percentile change and Bayley-III outcomes, although ubiquitously a trend was noted (Correlation Coefficients −0.18 to −0.12; Table 3).

**Table 3:** Unadjusted associations are presented as spearman rank correlation coefficients (*ρ*) with two-sided *p*-values. Adjusted *β* coefficients and *p*-values derive from multivariable linear regression models including gestational age, sex, sepsis, invasive mechanical ventilation duration, brain growth rate, brain volume at discharge, and head circumference percentile change.

Bayley domain/predictors		Correlation		Multivariable linear regression	
		Spearman *ρ*	*p*-value	Adjusted *β* (95% CI)	*p*-value
Cognitive	Brain Growth (cm3/d)	0.13	0.25	0.01 (−0.17–0.19)	0.91
	Brain Volume at Discharge (cm3)	−0.05	0.69	−0.02 (−0.09–0.05	0.64
	Head Circumference Percentile Drop	−0.15	0.19	−0.04 (−0.10–0.02	0.21
	Gestational Age	0.13	0.26	0.07 (−0.14–0.28)	0.51
	Sex	−0.04	0.71	−0.06 (−0.33–0.21)	0.66
	Sepsis	−0.19	0.09	−0.22 (−0.55–0.11)	0.19
	Mechanical Ventilation Duration	−0.18	0.11	**−0.33** **(−0.61 to −0.05)**	*
Language	Brain Growth (cm3/d)	0.17	0.13	0.11 (−0.13–0.35)	0.35
	Brain Volume at Discharge (cm3)	−0.06	0.61	−0.03 (−0.11–0.05)	0.39
	Head Circumference Percentile Drop	−0.12	0.28	−0.05 (−0.12–0.33)	0.16
	Gestational Age (weeks)	0.14	0.21	0.09 (−0.15–0.33)	0.45
	Sex	−0.05	0.67	−0.07 (−0.35–0.22)	0.64
	Sepsis (all kind)	−0.21	0.07	−0.26 (−0.53–0.02)	0.07
	Mechanical Ventilation Duration	−0.18	0.11	**−0.30** (**−0.58 to −0.01)**	*
Motor	Brain Growth (cm3/d)	**0**.**25**	*	0.27 (−0.09–0.63)	0.14
	Brain Volume at Discharge (cm3)	−0.0	0.98	−0.03 (−0.09–0.07)	0.83
	Head Circumference Percentile Drop	−0.18	0.11	−0.06 (−0.14–0.02)	0.14
	Gestational Age	0.21	0.06	0.18 (−0.06–0.43)	0.14
	Sex	−0.05	0.62	−0.09 (−0.38–0.20)	0.53
	Sepsis	**−0**.**30**	*	−0.23 (−0.50–0.04)	0.09
	Mechanical Ventilation Duration	−0.21	0.07	−0.19 (−0.43–0.06)	0.14
Combined	Brain Growth (cm3/d)	0.20	0.09	0.11 (−0.12–0.35)	0.34
	Brain Volume at Discharge (cm3)	−0.01	0.95	−0.01 (−0.08–0.06)	0.80
	Head Circumference Percentile Drop	−0.17	0.14	−0.05 (−0.12–0.03)	0.21
	Gestational Age	0.19	0.09	0.12 (−0.11–0.36)	0.30
	Sex	−0.05	0.62	−0.09 (−0.38–0.20)	0.53
	Sepsis	−0.25	0.02	−0.21 (−0.46–0.05)	0.11
	Mechanical Ventilation Duration	−0.19	0.09	**−0.29** (**−0.56 to −0.02)**	*

HC, head circumference.

*p < 0.05.

### Brain volume at discharge and neurodevelopment

Total brain volume at discharge was not significantly associated with Bayley-III percentile scores in any developmental domain. Correlation coefficients ranged from −0.06 to −0.0, with all *p*-values exceeding 0.61 (Table 3).

## Discussion

Our study investigated the relationship between sonographically assessed longitudinal brain volume growth and neurodevelopmental outcome at two years corrected age in very low birth weight (VLBW) preterm infants. The results demonstrate a statistically significant association between the rate of cerebral growth and motor function as assessed by Bayley-III percentile ranks. While the findings for cognitive and language domains did not reach statistical significance, bedside ultrasound can yield meaningful prognostic information on neurodevelopment.

The correlation between cerebral growth and motor outcome supports previous literature highlighting the predictive value of early brain development for motor function (13, 14, 20, 27, 36). In contrast to studies examining fetal brain volume in more stable prenatal conditions (37), the present analysis focuses on longitudinal postnatal brain growth, a dynamic process unfolding during a period of substantial physiological stress and medical intervention.

The strength of the association observed between brain growth rate and motor outcome in this cohort was modest, reflecting the complex and multifactorial nature of neurodevelopment in very low birth weight preterm infants. While the observed correlation between cerebral growth rate and motor outcome was statistically significant, a correlation of *r* = 0.25 explains only a modest proportion of outcome variance. The wide inter-individual overlap precludes reliable prediction at the single-patient level, and these findings should therefore be interpreted as hypothesis-generating within a comprehensive neurodevelopmental surveillance framework rather than as a standalone clinical decision tool. But the significance found in the motor domain aligns with the hypothesis that motor development may be more directly influenced by cerebral structures readily visualized by cranial ultrasound, such as the periventricular region, basal ganglia, and cerebellum (7, 8). The absence of significant correlations with cognitive and language domains suggests that these functions may rely more heavily on microstructural integrity and network maturation, aspects less directly captured by ultrasound-based volumetrics (10, 26). The moderate correlation observed between combined Bayley percentile score and cerebral growth approaches statistical significance and may suggest that brain growth, while an important marker, is insufficient as a standalone predictor of global neurodevelopment. The attenuation of the association between brain growth and motor outcome after adjustment suggests that cerebral growth reflects a shared pathway influenced by illness severity rather than an isolated causal factor. This is consistent with multivariate models in the literature that emphasize the need to account for other biological, environmental, and social factors (3, 11, 25). While cerebral growth alone is insufficient as a standalone predictor, its integration into multimodal risk models may enhance early identification of infants who benefit most from targeted neurodevelopmental surveillance. The absence of significant associations for cognitive and language outcomes underscores the complexity of higher-order neurodevelopment and the limitations of macrostructural imaging in capturing network-level maturation. Specifically, serial ultrasound-derived cerebral growth rates could complement established clinical risk markers—such as gestational age, duration of invasive ventilation, and somatic growth trajectories—in multimodal prediction models for early neurodevelopmental risk stratification. At the bedside, systematically tracked TBV trajectories could inform the timing of referrals for targeted follow-up or early intervention programs.

Interestingly, brain volume at discharge showed no significant association with any developmental of any functional domain. This finding reinforces the importance of analyzing brain growth trajectories over time rather than relying on a single volumetric measure at term-equivalent age, consistent with recent studies advocating for dynamic rather than cross-sectional brain monitoring (23, 24).

The study further revealed a relevant decline in body composition percentiles from birth to discharge, particularly in body weight, length, and head circumference. These findings mirror earlier observations that VLBW infants often fail to maintain intrauterine growth trajectories postnatally, despite overall gains in absolute measurements (21). The deceleration in head circumference percentile, while often used as a crude indicator of neurodevelopmental risk, is shown here to be significantly correlated with ultrasound-assessed brain growth but not to the Bayley-III outcomes. This supports the idea that brain development visualized by head circumference alone as solemn indicator can be confounded by extra-axial fluid, skull growth patterns, and measurement variability (19, 22, 38).

Our methodology offers a significant advancement by providing a reliable, safe, and repeatable bedside imaging tool for monitoring brain development in real time. ICC measurements confirm the high reproducibility of the ultrasound-based measurements used for brain volume estimation. MRI, while considered the gold standard for brain volumetry, is often impractical in the VLBW population due to the need for sedation, exposure to noise, body temperature control, transport out of the NICU and cost (23, 26). Moreover, serial MRI data across the neonatal period are rarely available, and most published MRI studies rely on a single measurement at term-equivalent age, limiting insight into developmental trajectories (25, 27). In contrast, our use of serial ultrasound assessments enables dynamic monitoring and risk stratification over time.

There are several limitations to this study. First, the retrospective design introduces inherent biases, including variability in ultrasound timing and image quality. Although standardized imaging protocols were used, inter-operator differences may have influenced measurement reliability, despite secondary review by a trained neonatologist. Second, while the ellipsoid formula used for estimating brain volume is validated (39), it remains an approximation and does not capture subtle regional changes or asymmetries. Third, the Bayley-III, while widely accepted, has been criticized for potentially underestimating developmental delays, particularly in language and cognitive domains. The use of percentile ranks and a combined score in this study was intended to enhance comparability and provide a global developmental perspective, but may have masked domain-specific deviations (33, 35). Furthermore, the relatively small cohort size limits generalizability and statistical power, particularly in subgroup analyses. Despite an initial cohort of 194 preterm neonates, only 79 infants had complete neurodevelopmental follow-up, representing a loss to follow-up of approximately 50% from the eligible cohort of 153 infants. This represents a significant limitation and a potential source of selection bias that warrants explicit acknowledgment. Infants lost to follow-up may disproportionately represent those at higher neurodevelopmental risk—for instance due to greater illness burden, social vulnerability, or logistical barriers to attendance—which could result in an overestimation of the associations observed in the analyzed cohort. Obtaining outcome data for these infants retrospectively is no longer feasible, as the cohort was born between 2019 and 2021 and the children are currently approximately four to six years of age; neither the PARCA-R questionnaire nor the Bayley-III are validated for use beyond 24 months corrected age, and alternative instruments available at this age would not be directly comparable to the existing outcome data. The sample size was sufficient to detect moderate associations; however, smaller effect sizes may not have been fully captured, particularly in gestational age subgroup analyses. Birth weight and small-for-gestational-age status were not included in the final multivariable models because of their close association with gestational age, which was retained as the primary maturity-related variable to reduce collinearity. In addition, necrotizing enterocolitis/focal intestinal perforation could not be reliably assessed as an independent predictor because only a small number of infants were affected in this cohort, limiting statistical power and model stability. Given the exploratory nature of secondary analyses across multiple developmental domains, results were interpreted with caution and emphasis was placed on consistency and effect direction rather than isolated *p*-values.

Finally, while the study focuses on the correlation between brain growth and outcome, causality cannot be inferred. Brain growth may reflect a constellation of factors including nutrition, systemic inflammation, respiratory support, and genetics—none of which were independently controlled for in this analysis. We therefore can underline the need for a multivariate approach in identifying variables for a prognosis of neurodevelopmental development.

## Conclusion

This study demonstrates that longitudinal brain volume growth, as assessed by serial cranial ultrasound, is significantly associated with motor developmental outcomes at two years corrected age in very low birth weight preterm infants. The findings support the clinical value of dynamic, bedside ultrasound as a feasible and informative tool for monitoring cerebral development in this vulnerable population. The ability to track individual brain growth trajectories over time offers an important advantage over single-point imaging approaches and may serve as an early indicator of neurodevelopmental risk. Future studies should aim to validate these findings in larger, prospective cohorts and explore integration with comprehensive clinical and biological risk models to optimize early identification and intervention strategies.

## Data Availability

The raw data supporting the conclusions of this article will be made available by the authors, upon reasonable request. Anonymized data are available in the Supplementary Materials.

## References

[1] Glass HC Costarino AT Stayer SA Brett CM Cladis F Davis PJ. Outcomes for extremely premature infants. Anesth Analg. (2015) 120(6):1337–51. 10.1213/ANE.0000000000000705 25988638 PMC4438860

[2] Han JH Yoon SJ Lim JH Shin JE Eun HS Park MS The impact of neonatal morbidities on child growth and developmental outcomes in very low birth weight infants: a nationwide cohort study. Eur J Pediatr. (2022) 181(1):197–205. 10.1007/s00431-021-04177-x 34236516

[3] Martini S Lenzi J Paoletti V Maffei M Toni F Fetta A Neurodevelopmental correlates of brain magnetic resonance imaging abnormalities in extremely low-birth-weight infants. J Pediatr. (2023) 262:113646. 10.1016/j.jpeds.2023.113646 37516269

[4] Brouwer MJ Kersbergen KJ van Kooij BJM Benders MJNL van Haastert IC Koopman-Esseboom C Preterm brain injury on term-equivalent age MRI in relation to perinatal factors and neurodevelopmental outcome at two years. PLoS One. (2017) 12(5):e0177128. 10.1371/journal.pone.0177128 28486543 PMC5423624

[5] Boardman JP Counsell SJ. Invited review: factors associated with atypical brain development in preterm infants: insights from magnetic resonance imaging. Neuropathol Appl Neurobiol. (2020) 46(5):413–21. 10.1111/nan.12589 31747472 PMC7496638

[6] Perlman JM. Neurobehavioral deficits in premature graduates of intensive care–potential medical and neonatal environmental risk factors. Pediatrics. (2001) 108(6):1339–48. 10.1542/peds.108.6.1339 11731657

[7] Dimitrova R Pietsch M Christiaens D Ciarrusta J Wolfers T Batalle D Heterogeneity in brain microstructural development following preterm birth. Cerebral Cortex. (2020) 30(9):4800–10. 10.1093/cercor/bhaa069 32306044 PMC7391275

[8] Trimarco E Jafrasteh B Jiménez-Luque N Marín Almagro Y Román Ruiz M Lubián Gutiérrez M Thalamic volume in very preterm infants: associations with severe brain injury and neurodevelopmental outcome at two years. Front Neurol. (2024) 15:1427273. 10.3389/fneur.2024.1427273 39206295 PMC11349527

[9] Arimitsu T Shinohara N Minagawa Y Hoshino E Hata M Takahashi T. Differential age-dependent development of inter-area brain connectivity in term and preterm neonates. Pediatr Res. (2022) 92(4):1017–25. 10.1038/s41390-022-01939-7 35094022 PMC9586860

[10] Dimitrova R Arulkumaran S Carney O Chew A Falconer S Ciarrusta J Phenotyping the preterm brain: characterizing individual deviations from normative volumetric development in two large infant cohorts. Cerebral Cortex. (2021) 31(8):3665–77. 10.1093/cercor/bhab039 33822913 PMC8258435

[11] Sammallahti S Pyhälä R Pesonen A-K Heinonen K Hovi P Eriksson JG Infant growth after preterm birth and neurocognitive abilities in young adulthood. J Pediatr. (2014) 165(6):1109–15.e3. 10.1016/j.jpeds.2014.08.028 25262301

[12] Burkitt K Kang O Jyoti R Mohamed A-L Chaudhari T. Comparison of cranial ultrasound and MRI for detecting BRAIN injury in extremely preterm infants and correlation with neurological outcomes at 1 and 3 years. Eur J Pediatr. (2019) 178(7):1053–61. 10.1007/s00431-019-03388-7 31065842

[13] Beunders VAA Roelants JA Suurland J Dudink J Govaert P Swarte RMC Early ultrasonic monitoring of brain growth and later neurodevelopmental outcome in very preterm infants. Am J Neuroradiol. (2022) 43(4):639–44. 10.3174/ajnr.A7456 35332022 PMC8993199

[14] Zhang XH Qiu S-J Chen W-J Gao X-R Li Y Cao J. Predictive value of cranial ultrasound for neurodevelopmental outcomes of very preterm infants with brain injury. Chin Med J (Engl). (2018) 131(8):920–6. 10.4103/0366-6999.229895 29664051 PMC5912057

[15] Wu YW Monsell SE Glass HC Wisnowski JL Mathur AM McKinstry RC How well does neonatal neuroimaging correlate with neurodevelopmental outcomes in infants with hypoxic-ischemic encephalopathy? Pediatr Res. (2023) 94(3):1018–25. 10.1038/s41390-023-02510-8 36859442 PMC10444609

[16] Benavente-Fernández I Ruiz-González E Lubian-Gutiérrez M Lubián-Fernández SP Cabrales Fontela Y Roca-Cornejo C Ultrasonographic estimation of total brain volume: 3D reliability and 2D estimation. Enabling routine estimation during NICU admission in the preterm infant. Front Pediatr. (2021) 9:708396. 10.3389/fped.2021.708396 34368031 PMC8339409

[17] Cuzzilla R Spittle AJ Lee KJ Rogerson S Cowan FM Doyle LW Postnatal brain growth assessed by sequential cranial ultrasonography in infants born<30 Weeks’ gestational age. AJNR Am J Neuroradiol. (2018) 39(6):1170–6. 10.3174/ajnr.A5679 29773561 PMC7410612

[18] Stein A Sody E Bruns N Felderhoff-Müser U. Development of an ultrasound scoring system to describe brain maturation in preterm infants. AJNR Am J Neuroradiol. (2023) 44(7):846–52. 10.3174/ajnr.A7909 37321856 PMC10337624

[19] Dieks J-K Jünemann L Hensel KO Bergmann C Schmidt S Quast A Stereophotogrammetry can feasibly assess ‘physiological’ longitudinal three-dimensional head development of very preterm infants from birth to term. Sci Rep. (2022) 12(1):8940. 10.1038/s41598-022-12887-x 35624305 PMC9136805

[20] Brickmann C Lampe R Sidorenko I Gauger N Hauer J Krüger M Sonographic brain volume growth trajectories in VLBW and clinical determinants—data from the NeoNEVS project. Children. (2026) 13(2):281. 10.3390/children13020281 41749637 PMC12939424

[21] Rochow N Raja P Liu K Fenton T Landau-Crangle E Göttler S Physiological adjustment to postnatal growth trajectories in healthy preterm infants. Pediatr Res. (2016) 79(6):870–9. 10.1038/pr.2016.15 26859363

[22] Ranke MB Krägeloh-Mann I Vollmer B. Growth, head growth, and neurocognitive outcome in children born very preterm: methodological aspects and selected results. Dev Med Child Neurol. (2015) 57(1):23–8. 10.1111/dmcn.12582 25251724

[23] Romberg J Wilke M Allgaier C Nägele T Engel C Poets CF MRI-based brain volumes of preterm infants at term: a systematic review and meta-analysis. Arch Dis Childhood Fetal Neonatal Ed. (2022) 107(5):520–6. 10.1136/archdischild-2021-322846 35078779 PMC9411894

[24] Ruiz-González E Lubián-López SP Jiménez Luque N Segado-Arenas A Lubián-Gutiérrez M Almagro YM Relationship of early brain growth pattern measured by ultrasound with neurological outcome at two years of age in very low birth weight infants. Eur J Pediatr. (2023) 182(11):5119–29. 10.1007/s00431-023-05170-2 37682341 PMC10640451

[25] Medina-Alva P Duque KR Zea-Vera A Bellomo S Cárcamo C Guillen-Pinto D Combined predictors of neurodevelopment in very low birth weight preterm infants. Early Hum Dev. (2019) 130:109–15. 10.1016/j.earlhumdev.2019.01.019 30743197 PMC6478608

[26] Dubois M Legouhy A Corouge I Commowick O Morel B Pladys P Multiparametric analysis of cerebral development in preterm infants using magnetic resonance imaging. Front Neurosci. (2021) 15:658002. 10.3389/fnins.2021.658002 33927592 PMC8076519

[27] Selvanathan T Guo T Kwan E Chau V Brant R Synnes AR Head circumference, total cerebral volume and neurodevelopment in preterm neonates. Arch Dis Child Fetal Neonatal Ed. (2022) 107(2):181–7. 10.1136/archdischild-2020-321397 34261769

[28] Yin J Wu Y Shi Y Shen L Yin Q. Relationship between the quantitative indicators of cranial MRI and the early neurodevelopment of preterm infants. Comput Math Methods Med. (2021) 2021:6486452. 34840597 10.1155/2021/6486452 PMC8626187

[29] Hou W Tang PH Agarwal P. The most useful cranial ultrasound predictor of neurodevelopmental outcome at 2 years for preterm infants. Clin Radiol. (2020) 75(4):278–86. 10.1016/j.crad.2019.11.009 31870490

[30] Tang L Xiao F Gao Y Zhang P Cheng G Wang L Neurosonography: shaping the future of neuroprotection strategies in extremely preterm infants. Heliyon. (2024) 10(11):e31742. 10.1016/j.heliyon.2024.e31742 38845994 PMC11154624

[31] Boecker H Scheef L Jankowski J Zimmermann N Born M Heep A. Current stage of fMRI applications in newborns and children during the first year of life. Rofo. (2008) 180(8):707–14. 10.1055/s-2008-1027483 18642213

[32] Benavente-Fernández I Rodríguez-Zafra E León-Martínez J Jiménez-Gómez G Ruiz-González E Fernández-Colina RC Normal cerebellar growth by using three-dimensional US in the preterm infant from birth to term-corrected age. Radiology. (2018) 288(1):254–61. 10.1148/radiol.2018171956 29613844

[33] Torres-González C Ricardo-Garcell J Alvarez-Núñez D Galindo-Aldana G. Intellectual development in Mexican preterm children at risk of perinatal brain damage: a longitudinal study. Children. (2024) 11(6):652. 10.3390/children11060652 38929232 PMC11201988

[34] Kline JE Illapani VSP He L Altaye M Logan JW Parikh NA. Early cortical maturation predicts neurodevelopment in very preterm infants. Arch Dis Childhood Fetal Neonatal Ed. (2020) 105(5):460–5. 10.1136/archdischild-2019-317466 31704737 PMC7205568

[35] Hintz SR Vohr BR Bann CM Taylor HG Das A Gustafson KE Preterm neuroimaging and school-age cognitive outcomes. Pediatrics. (2018) 142(1):e20174058. 10.1542/peds.2017-4058 29945955 PMC6128951

[36] Montgomery C Setänen S Kaul YF Farooqi A Broström L Aden U Predictive value of bayley-III motor Index for later motor difficulties in children born extremely preterm. Acta Paediatr. (2023) 112(4):742–52. 10.1111/apa.16694 36723223

[37] Sadhwani A Wypij D Rofeberg V Gholipour A Mittleman M Rohde J Fetal brain volume predicts neurodevelopment in congenital heart disease. Circulation. (2022) 145(15):1108–19. 10.1161/CIRCULATIONAHA.121.056305 35143287 PMC9007882

[38] Miyabayashi H Nagano N Kato R Hashimoto S Saito K Noto T Cranial shapes of Japanese preterm infants at one month of age using a three-dimensional scanner. Brain Dev. (2022) 44(10):690–8. 10.1016/j.braindev.2022.07.004 35906116

[39] Nguyen The Tich S Anderson PJ Shimony JS Hunt RW Doyle LW Inder TE. A novel quantitative simple brain metric using MR imaging for preterm infants. AJNR Am J Neuroradiol. (2009) 30(1):125–31. 10.3174/ajnr.A1309 18832662 PMC2818625

