# Supplementary material for: The relationship between sonographically assessed volumetric brain development in VLBW preterm infants and neurodevelopmental outcome at 2 years of age—data from the NeoNEVS project
Source: Front Pediatr. 2026 Apr 13;14:1769403. doi: 10.3389/fped.2026.1769403 (PMC13111570; doi:10.3389/fped.2026.1769403)
Supplement: Supplementary file 1 [file Table1.docx]

**Supplement**

| Measurements | Interrater ICC (CI 95) | Intrarater ICC (CI 95) |
| --- | --- | --- |
| Biparietal Coronar | 0.965 (0.939-0.979) | 0.975 (0.961-0.984) |
| Sagittal anterio-posterior | 0.971 (0.955-0.981) | 0.978 (0.965-0.986) |
| Sagittal Vertical | 0.935 (0.870-0.964) | 0.943 (0.911-0.963) |
| TBV | 0.975 (0.938-0.987) | 0.980 (0.967-0.988) |

Tab. 1: Intra- and Interrater Reliability Measurements of 15 patients covering the three sonographic planes and the resulting TBV. Abr.: ICC=Interclass Correlation Coefficient; CI=Confidence Interval; TBV=Total Brain Volume.
